# Exploring the trajectory of post-traumatic growth in patients in intensive care unit: a phenomenological longitudinal study

**DOI:** 10.1080/17482631.2025.2535838

**Published:** 2025-07-20

**Authors:** Hongyi Li, Xue Li, Yuying Fan, Jingshu Li, Xiaona Qi

**Affiliations:** a Clinical Nursing Teaching and Research Office, Second Affiliated Hospital of Harbin Medical University, Harbin, China; b School of Nursing, Harbin Medical University, Harbin, China; c Department of Nursing, The Cancer Hospital of Harbin Medical University, Harbin, China

**Keywords:** Intensive care unit, post-traumatic growth, longitudinal study, qualitative studies, psychology nursing, phenomenology, ICU outcomes

## Abstract

**Purpose:**

Post-traumatic growth (PTG) has been increasingly observed in various trauma groups. However, few studies have longitudinally explored PTG in intensive care unit (ICU) patients using qualitative methods. This study aimed to explore PTG pathways in ICU patients and offer reference data and methodological insights for personalized interventions.

**Methods:**

A phenomenological longitudinal study was conducted among patients transferred from ICU between May 2023 and February 2024. Four interview points were determined: Day two after transfer to ICU (T0), the 1st month after transfer out of ICU (T1), the 3rd month after transfer out of ICU (T2), and the 6th month after transfer out of ICU (T3).

**Results:**

21 cases were included. Two PTG trajectories were identified: “sublimation PTG trajectory” and “compound dual-path PTG trajectory,” comprising four periods: “trauma response,” “growth transition,” “collapse,” and “transition recovery.” Fifteen themes emerged, including “the persistent presence of avoidance responses,” with details shown in figures and tables.

**Conclusions:**

PTG in ICU patients showed shared and individualized patterns. Clinicians should recognize key time points, support self-healing, and tailor extended care. A multidisciplinary aftercare model combining online and in-person services is recommended for long-term recovery.

## Introduction

With the remarkable advancement of modern medical technology, the survival and long-term recovery rates of patients in Intensive Care Units (ICUs) have significantly improved (Doherty et al., 2022; Weissman et al., 2020). Nevertheless, these medical advances have not resolved the persistent and multifaceted health challenges faced by ICU survivors. Studies have shown that a subset of ICU survivors develop new or worsening impairments in physical, cognitive, or mental health that persist beyond acute hospitalization, collectively referred to as Post-Intensive Care Syndrome (PICS) (Needham et al., 2012). The incidence of psychological disorders—such as post-traumatic stress disorder (PTSD), depression, and anxiety—has been reported to range from 22% to 46% among ICU survivors, significantly higher than that observed in the general population (Hatch et al., 2018). Longitudinal studies have indicated that approximately one-third of ICU survivors continue to exhibit significant psychological symptoms one year after discharge (Bastian et al., 2018). These symptoms are often chronic (Teixeira et al., 2021), rarely remit spontaneously (Myhren et al., 2010), and significantly compromise both quality of life and social reintegration (Bryant & McNabb, 2019; Colbenson et al., 2019), while also substantially increasing the risk of self-harm and suicide (Fernando et al., 2021). These issues have become an urgent and unresolved clinical challenge within critical care medicine.

In recent years, research on the psychological health of ICU patients has expanded, but most studies have been grounded in a negative psychological perspective, focusing primarily on adverse outcomes such as PTSD, depression, and anxiety. While this perspective has provided valuable insights into the mental health challenges faced by ICU survivors, it remains limited in its theoretical development and intervention design, often failing to capture the dynamic processes of psychological recovery. With the rise of positive psychology, growing scholarly interest has focused on the transformative potential of trauma.This shift has encouraged a more holistic understanding of psychological adaptation in the aftermath of critical illness. In particular, the theory of Post-Traumatic Growth (PTG), proposed by Tedeschi and Calhoun in 1996, offers a robust framework for understanding positive psychological changes that may occur in the aftermath of severe trauma. PTG describes a process whereby individuals engage in cognitive restructuring and psychological adaptation, leading to growth that surpasses their pre-trauma functioning. Its five core domains include enhanced personal strength, improved interpersonal relationships, newly perceived life possibilities, a deepened appreciation for life, and transformed self-perception (Tedeschi & Calhoun, 1995).This theory has received empirical support across diverse populations—including cancer survivors (Di et al., 2023), caregivers of individuals with chronic illness (Lin et al., 2021), and survivors of natural disasters—and has provided important theoretical guidance for psychosocial interventions targeting trauma-affected groups.

Despite its demonstrated relevance across various trauma contexts, PTG remains underexplored in ICU populations, particularly in terms of its longitudinal development. The ICU treatment is highly specific in nature, exposing patients to distinctive trauma-related stressors—such as imminent life threats, prognostic uncertainty, and invasive procedures—which may fundamentally alter their psychological response mechanisms, setting them apart from other trauma-exposed populations (Horowitz, 1986). Therefore, the direct application of existing PTG frameworks may be insufficient to fully capture the distinctive features of psychological recovery among ICU survivors and may provide limited practical guidance for tailoring individualized interventions.Against this backdrop, there is an urgent need for systematic and in-depth longitudinal research that explores the unique trajectories of posttraumatic growth (PTG) among ICU survivors. Such research may help promote a shift in ICU psychological intervention strategies—from a sole focus on alleviating negative emotional states to embracing a more holistic philosophy that emphasizes the facilitation of positive psychological growth. Accordingly, this study aimed to investigate how individuals psychologically adapt and grow in the aftermath of intensive care, and how they construct personal meaning within the distinctive context of the ICU experience.

## Materials and methods

### Objects of the study

**Figure 1.**
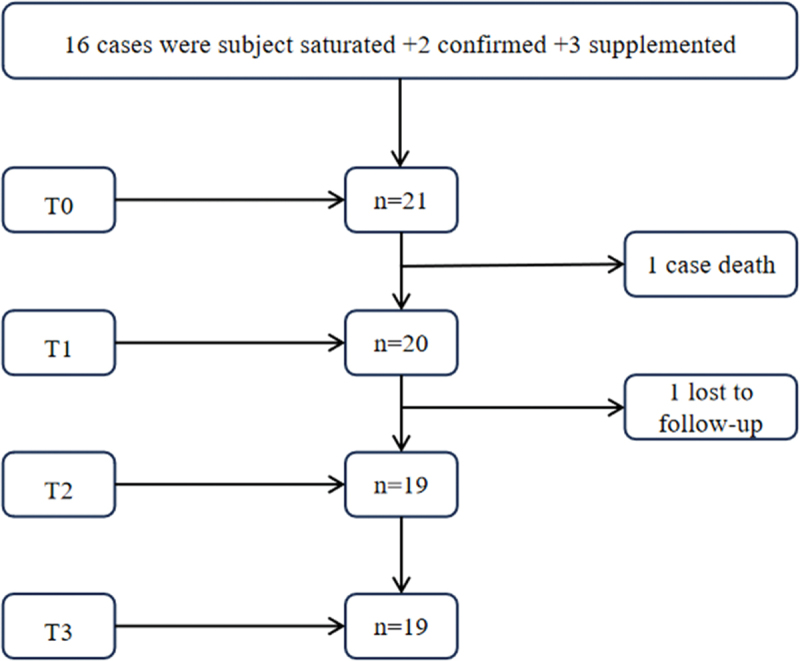
The number of interviewees included and loss.

This study adopted purposive sampling combined with the principle of maximum variation, aiming to maximize sample heterogeneity. Informed by the research question and relevant literature, variation was defined in terms of heterogeneity in participants’ sociodemographic characteristics (e.g., age, gender, educational background, marital status) and clinical profiles (e.g., type of illness, mode of admission, length of ICU stay). To systematically implement the sampling strategy, the research team developed a sampling matrix prior to recruitment, which outlined representative categories across key sociodemographic and clinical dimensions. During the recruitment process, the distribution of participants across these categories was continuously monitored. When certain categories were underrepresented, the team collaborated with clinical staff to conduct targeted supplementary recruitment, ensuring structural coverage of core variables. This approach was intended to enhance the conceptual richness and theoretical transferability of the study findings. Patients who were treated in and subsequently transferred out of the ICU at the Second Affiliated Hospital of Harbin Medical University between May 2023 and February 2024 were selected as study participants. Inclusion criteria were: age ≥18 years; ICU stay of at least 48 hours; no recent experience of major life events (e.g., natural disasters, serious traffic accidents, bereavement, or unemployment); and provision of informed consent with voluntary participation. Exclusion criteria included: a history of cognitive impairment or mental illness; terminal cancer; a poor prognosis; receipt of palliative care; or dysarthria/communication difficulties due to medical treatment. Participants were excluded if they died during follow-up, were lost to follow-up (defined as two consecutive unsuccessful contact attempts post-ICU discharge), or declined to continue participation. The study was approved by the Ethics Committee of the Second Affiliated Hospital of Harbin Medical University (Approval No. YJSKY2023–039), and all participants provided informed and voluntary consent. The sample size at the first time point was determined based on the principle of data saturation (Zhang & Chen, 2024), with two additional participants added for further validation (Yang et al., 2022). To account for potential sample attrition, three more participants were recruited as supplements (Kneck & Audulv, 2019), as shown in Figure 1. A total of 21 participants were ultimately identified and included in this study, labelled P1 to P21. In total, 79 interviews were conducted. General demographic and clinical information of the participants is presented in Table I.

**Table I.** The characteristics of interviewees (*n* = 21).

No.	Age (years)	Sex	Disease diagnosis	Literacy	Marital status	Occupation	Length of stay (days)	Religion
P1	59	male	Diabetic ketoacidosis	Junior high	Divorced	Freelancing	10	Islam
P2	36	female	Heavy bleeding in the upper digestive tract	College	Married	Employee	8	no
P3	56	female	Cirrhosis decompensation	Senior high school	Married	Unemployed	5	no
P4	60	female	Acute kidney failure	College	Married	Retirees	8	no
P5	23	female	Diabetic ketoacidosis	Junior high	Married	Employee	6	no
P6	54	male	Heavy bleeding in the upper digestive tract	Junior High	Married	Peasant	6	Buddhism
P7	62	female	Hydronephrosis	High school	Married	Retired workers	7	Christian
P8	68	male	Secondary epilepsy	High school	Married	Freelancing	6	no
P9	47	male	Chronic kidney failure	Graduate students	Married	Engineer	7	no
P10	43	female	Acute kidney failure	High school	Divorced	Employees	15	no
P11	79	male	Urinary tract infection	Graduate student	Married	Retired teachers	6	no
P12	29	male	Diabetic ketoacidosis	College	unmarried	Freelancing	5	no
P13	44	male	Severe acute pancreatitis	College	Married	Employee	7	no
P14	38	female	Severe acute pancreatitis	College	Divorced	worker	8	no
P15	53	female	Heavy bleeding in the upper digestive tract	High School	Married	Beautician	7	no
P16	19	male	Rhabdomyolysis	College	unmarried	Student	18	no
P17	56	male	septic shock	Junior high school	Married	Farmer	9	no
P18	33	male	Diabetic ketoacidosis	High School	unmarried	Unemployed	9	Buddhism
P19	43	male	Severe acute pancreatitis	Senior high school	Married	Freelancing	10	no
P20	41	male	Acute peritonitis	Junior high school	Married	Worker	6	no
P21	54	female	Respiratory failure	Elementary school	Married	Farmer	10	no

### Research design

This study employed a longitudinal qualitative research (LQR) design grounded in Interpretative Phenomenological Analysis (IPA) (Chen, 2000; Snelgrove, 2014). IPA aims to explore how individuals make sense of their lived experiences within specific contexts, making it suitable for examining how patients construct meaning during their recovery. LQR involves collecting qualitative data from the same participants at two or more time points, enabling an in-depth exploration of their evolving trajectories over time (Saldana, 2003). As LQR methodologies continue to evolve (Chen & Zhou, 2022), scholarly attention has extended beyond intra-individual temporal variation to include the temporal mapping of divergent trajectories across individuals. The integration of IPA and LQR facilitates a systematic portrayal of the evolving process of post-traumatic growth (PTG) among ICU survivors within a specific medical context, by capturing both temporal dynamics and the construction of meaning over time.

To ensure the scientific rigour and practical relevance of the interview protocol and time-point design, an initial framework was constructed based on a review of relevant literature (Bryant & McNabb, 2019; Zhang et al., 2024).This framework was further refined through clinical observations, group discussions, and expert consultations. Specifically, the literature review provided the theoretical foundation and structural framework; clinical observations ensured that the interview content resonated with real-world ICU settings; group discussions were used to refine the logical coherence and linguistic formulation of the questions; and expert consultations provided guidance on enhancing the scientific integrity, normative alignment, and clinical applicability of the content.After the preliminary development of the interview guide, two rounds of pilot interviews were conducted and revised based on feedback from participants. The final version of the guide was then reviewed and modified by a multidisciplinary panel of experts, including specialists in ICU medicine, nursing, psychology, and interpersonal communication. This review process enhanced the differentiation and coherence of the questions across the various time points, ensuring that the interview content was both theoretically robust and clinically meaningful. Four interview points were finally determined: Day two after transfer to ICU (T0), the 1st month after transfer out of ICU (T1), the 3rd month after transfer out of ICU (T2), and the 6th month after transfer out of ICU (T3). Face-to-face interviews were conducted with the respondents in the ICU at T0, while personal communication were conducted at T1, T2, and T3.

### Data collection methods

Data were collected through semi-structured interviews. Before each interview, the researchers conducted a comprehensive assessment of the participant’s medical condition and treatment context to ensure both safety and suitability for participation. Efforts were made to establish rapport and provide humanistic care, thereby fostering trust between the researcher and the participant. Participants were thoroughly informed about the purpose, content, and significance of the interview, and were assured of confidentiality and voluntary participation. Informed consent was obtained prior to the full audio-recording of the interview. Throughout the interviews, researchers engaged in active and empathetic listening, while also observing and documenting non-verbal cues such as facial expressions and eye movements. The interview guide was applied flexibly, allowing for modifications to the question order based on the participant’s responses. Privacy and confidentiality were strictly maintained throughout the process. Each interview lasted approximately 30–45 minutes, and audio recordings were transcribed verbatim within 12 hours of completion. The transcripts were then double-checked by two researchers for accuracy. Table II presents the detailed interview guide, including its structure and format.

**Table II.** Outline and form of interview.

Timing	Interview outline	Format
T0	① Can you talk about the treatment experience in ICU these days? ② How do you understand this experience? Has any of these understandings changed? ③ Do you think there was any significant change before and after entering ICU? ④ What are your expectations and concerns about life after ICU transfer? What support do you need most now?	Face to face Interviews
T1	① Have you gained a new understanding of your ICU experience since the last interview? ② How have you changed your understanding of yourself since you were in the ICU? ③ Have you encountered any challenges in this month? How to overcome it? Do you need any support?	Telephone Interviews
T2	① Has the ICU experience affected your current life? ② Compared with before, what do you think is the most important psychological change? ③ How is the current recovery different from what you expected? 4) Did you discover new abilities or encounter new difficulties during the process of rehabilitation?	
T3	① Looking back on the experience of ICU treatment, what do you feel most about now? ② How has this experience affected your attitude towards life? ③now will this experience affect your future life choices?	

### Data analysis

The data were primarily analysed manually, with the assistance of NVivo software. Given that longitudinal qualitative research (LQR) does not follow a standardized analytical approach (Saldana, 2003), this study adopted a combined framework of repeated cross-sectional and longitudinal analysis (SmithBattle et al., 2018; Tuthill et al., 2020). The specific steps for cross-sectional Interpretative Phenomenological Analysis are outlined as follows (Chen, 2000): (i) The researchers repeatedly read and immersed themselves in the data to gain a deep familiarity with the ICU patients’ narratives, identifying key phrases and meaning-laden segments related to their psychological experiences. (ii) Building on the suspension of preconceptions, the researcher immersed in understanding the meaning of the text within the patient’s life context, extracting initial meaning units; (iii) Through continuous induction and comparison, the researcher identified the internal relationships between these meaning units, abstracting and forming core themes and their hierarchical structure to reveal the essential characteristics of psychological experiences. The analytical process followed a cyclical and progressive strategy, refining initial interpretations, and deepening understanding through reflective writing and team discussions. When no new meaning units emerged, the cross-sectional analysis was considered complete, and the study progressed to the longitudinal analysis phase.

Regarding the longitudinal analysis, we employed a “temporal pooling” strategy (Saldana, 2003), wherein qualitative data from three time points (T1 to T3) were consolidated into a unified temporal pool. This enabled a trajectory-based exploration of thematic evolution across time, revealing how patients’ psychological experiences unfolded dynamically. The focus was placed on the continuity, changes, and reconstruction of specific themes across different time points, thereby mapping the trajectory of psychological transformation. At T3, a comprehensive retrospective analysis was conducted to further trace the long-term psychological impact of the ICU experience and the construction of personal meaning. This culminated in the development of typified trajectories of post-traumatic psychological growth among different groups of ICU patients. The entire analytical process adhered to the fundamental principles of interpretative analysis, emphasizing a deep interpretation of subjective experiences. It sought to explore how patients understood and integrated their ICU experiences within their lived worlds, while also presenting their life experiences and the process of meaning-making.

Data collection involved in-depth interviews, field observations, and document analysis (see Table III for details), ensuring the authenticity and rigour of the findings.Throughout the research process, the researchers engaged in reflective practice, setting aside preconceived assumptions and professional biases in order to understand participants’ experiences with openness and respect. During data collection and analysis, triangulation was employed to enhance the study’s credibility. Two full-time master’s students in nursing, who had received professional training, independently coded the data and engaged in regular discussions. Member checking was conducted for preliminary findings to ensure the trustworthiness of the results.Preliminary findings were verified through member checking. After analysing the objective data from each time point, feedback was promptly provided to participants for verification, ensuring the accuracy of the results. Data collection continued until no new themes emerged, at which point thematic saturation was achieved.

**Table III.** Original data collection methods and their applications.

Method	Practical Application	Significance
In-depth Interviews	Multiple rounds of semi-structured interviews were conducted to explore participants’ subjective experiences during ICU treatment, including their physical sensations, emotional responses, and cognitive changes.	Exploring first-person, in-depth experiences to understand how individuals construct meaning in their lived worlds.
Field Observation	Systematic capture of non-verbal communication, clinician–patient interactions, and environmental responses within clinical contexts.	Compensating for the limitations of verbal expression and enhancing the interpretation of subjective accounts.
Document Analysis	Review of existing ICU nursing research, presentation of policy recommendations, and establishment of a theoretical framework within the relevant background context.	Provision of an analytical framework to ensure scientific rigor and promote forward-thinking research.

ICU, intensive care unit.

Reducing Respondent Attrition: Longitudinal studies must effectively minimize sample loss to ensure research quality. The researchers obtained multiple contact information from the respondents and their families and an additional case-specific telephone follow-up 1 week after the patient was transferred out of the ICU, which provided unpaid post-hospital guidance throughout the study to improve respondent attrition.

## Results

**Figure 2.**
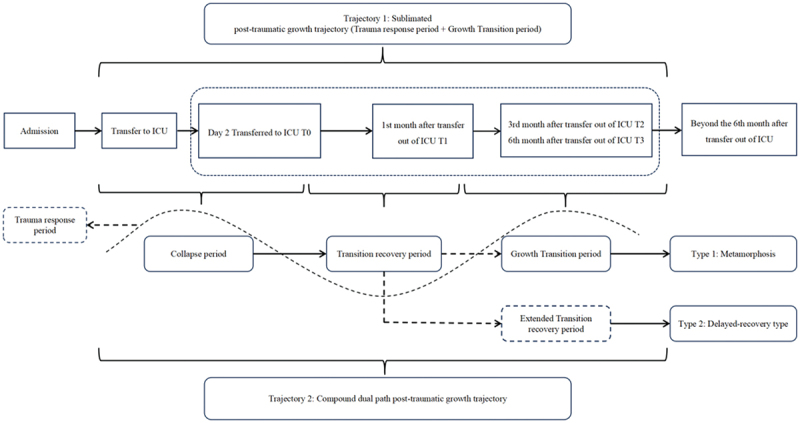
Journey trajectory of post-traumatic growth in patients in ICU.

This study explored two patterns of post-traumatic growth (PTG) trajectories among patients who had been in the intensive care unit (ICU). The findings revealed that a portion of patients followed a relatively stable trajectory (Trajectory 1), characterized as a sublimation-type post-traumatic growth trajectory, which primarily involved distinct phases of trauma response period and growth transition period. In contrast, a larger number of patients exhibited a more complex pattern (Trajectory 2), a compound dual path PTG trajectory dominated by metamorphic or delayed-growth types, in which trauma responses persisted intermittently throughout the process. Both trajectories involved phases of traumatic response period and growth transition period, although their intensity and expression varied.Detailed illustrations of the two trajectories are provided in Figure 2, with representative cases summarized in Table IV.

**Table IV.** Trajectory-period-themes-instance table.

Trajectory	Periods	Theme	Example citations
Trajectory 1: Sublimation PTG trajectory	Trauma response period	The persistent presence of avoidance responses	P12 (T0): “Don’t want to talk about it, just want to get better soon.” P1 (T2): “I don’t talk about the ICU. It’s just a worry.” P18 (T3): “There is no point in thinking about the past, I am recovering so well, one has to move on.”
		Positive Adjustment Self-awareness	P16 (T2): “Every once in a while, I was in the ICU, someone was rescuing me, or I wasn’t. Life is fragile. It’s not easy to live. We should cherish it.” P7 (T0): “Brothers and sisters in the church all wrote to encourage me, the Lord is bless me, sent so many angels to save me, I did not know that I was so strong.”
		Derived altruistic behaviour	P3 (T0): “When I came in, I thought, if I really can’t do it this time, I can donate all the organs I can use on my body, so that I can be of some use to society.” P18 (T1): “Now I often advise my friends around me to take good care of their bodies. They are still so young, don’t follow my old path. Cherish your health while you are healthy.”
		Changing their philosophy	P10 (T0): “This illness was sent to me by my ex-husband, and we had little contact after the divorce.” I used to feel that I had accumulated grudges in my heart, but now I think about it, what are these in the face of life and death? I am grateful to him for helping me.” P3 (T0): “I am very grateful to everyone for helping me. Last night when I was rescuing the next bed, I kept praying in my heart that the Buddha would bless him as he blessed me.”
		Information needs presented Stage characteristics	P9 (T0): “I want to know what my creatinine level is now, has it dropped? What kind of treatment should I use? What can be achieved after treatment?” P14 (T2): “Blood sugar seems to be a little low the other day, do you need to check it again? Or take some medicine?”
	Growth transition period	Behaviour-led Self-growth	P20 (T1): “I used to be a big drinker. Going to the ICU was a wake-up call for me, and I stopped drinking as soon as I left the hospital.” P11 (T3): “Now a person at home reading a book, see tired play online games, but also have to water the flowers, very busy, ha ha.”
		New self repair Needs	P2 (T2): “I probably need some time to myself, and slowly it should get better and life can completely get back to normal.” P14 (T3): “Now I want to try to cope on my own, I don’t want to bother people anymore, I just want people to see me as a normal person.”
Trajectory 2 Compound dual-path PTG trajectory	Collapse period	Built-in cognition Broken	P16: “I used to get sick easily, and I had to stay in the hospital for 10 days and a half a month each time, but I never got sick so quickly and seriously that I was sent directly to the ICU. I still haven’t had a reprieve.” P4: “I couldn’t wear clothes. When the doctors and nurses checked my skin after shift, I was very resistant and felt no dignity. The treatment was totally different from what I had imagined!”
		Trapped in intertwined and overwhelming negative emotions	P2: “I’m scared. I get nervous when the machine goes off. I’m only in my thirties. My baby is so small. I don’t want to die (sobbing).” P11: “I suddenly had a fever last night, and now my strength is so weak that I can hardly get up from bed. I am worried about whether I can take care of myself after I leave the hospital.” P13: “Many of the instruments here are quite expensive to use. After I fell ill, my family basically had no source of income, so I was worried.”
		Negative stress response Hair-trigger reaction	P4: “I don’t want to stay in ICU anymore. I don’t want to be treated. It’s too painful.” P8: “I was in protective restraints when I woke up and didn’t even ask, it wasn’t fair to me!” Today the nurse gave me fluids and I had to hide my hands and not cooperate.”
		Psychological needs Increased	P8: “When I came in my grandson kept saying ‘Grandpa don’t sleep in there all the time, come out early, don’t stay too long’, I want to see my family.” P11: “I really need the comfort of the medical staff, even if it is just a few words to reassure me not to worry, it will make me feel better.”
	Transition recovery period	Residual mind and body Health disturbance	P17: “After being transferred out of ICU, I obviously feel that my limbs are not as strong as before. Now I can’t even hold a child of 30 kilograms, and I am tired every move. It has been a month, and it is not good.” P1: “Coming back this month, I often have nightmares at night, more than when I was in ICU, always dreaming that the doctor at my bedside asked me to amputate and amputate my limbs.” (About 16 patients in this study repeatedly mentioned being troubled by nightmares)
		Hindered reintegration	P21: ‘My daughter won’t let me go out for a walk when I want to, she says she gets scared when I go out. She also said that my immunity is poor, and I am not allowed to talk to my neighbors, and I have to wear a mask to avoid people when I go out, like a thief.” P19: “After I was discharged from the hospital, I felt a little uncomfortable and ran to the hospital. I was very afraid. I always felt that my illness was not yet cured, and I dared not do anything.
		Sense of self-identity Impairment	P6: “Now, I am useless, I despise myself, I am a waste who can only spend money.” P7: “When I was in ICU, my symptoms (frequent urination and pain) were not so serious. I think they were aggravated by the poor care I took after discharge.” P13: “I used to handle everything at home, now…… (silence for 10 seconds), I’m of no use, I rely on her (lover) to support myself.”
		Optimistic Looking forward to recovery	P5: “Now the blood sugar control is very good, the doctor said in the review that if this trend continues, I will gradually be able to stop taking insulin and change to drug control.” P16: “Now my physiological indicators are normal, I should be able to go back to school soon.”

T0: Day two after transfer to ICU.

T1: The 1st month after transfer out of ICU.

T2: The 3rd month after transfer out of ICU.

T3: The 6th month after transfer out of ICU.

### Trajectory 1: sublimation PTG trajectory

The boundary between “quality” and “quantity” was clearly defined in the definition of PTG. It could be observed that PTG was both a course and a result. In this study, a minority of patients showed positive, concurrent cognitive, and behavioural growth from the early stage of ICU treatment; their adaptation level was far higher than that in patients in the same period. However, longitudinal observations showed that these patients only displayed various characteristics during the trauma response, growth, and transformation periods, with PTG remaining in a high and stable state. The level of adaptation was significantly improved in multiple ways, including philosophy of life and sublimation. These patients were often relatively young and highly educated, received timely rescue and recovery in the ICU after acute onset, and had relatively stable social support

### Trauma response period

The traumatic response period refers to the period after ICU treatment when patients experience a range of psychological reactions, such as persistent avoidance and active cognitive restructuring. The psychological features of this phase are embedded in both trajectories and persist throughout the entire recovery process. In Trajectory 1, the traumatic response period unfolds alongside the growth transition period, forming an integrated and mutually reinforcing process. In contrast, in Trajectory 2, the traumatic response phase emerges earlier and continues to interweave with the entire process, playing a role in facilitating transformation. While this phase reveals shared psychological patterns among patients, it manifests with greater depth, nuance, and complexity in Trajectory 1 than in Trajectory 2.

The experience of the ICU often enables patients to gain a deeper understanding about the fragility of life; appreciate and cherish life; motivate themselves for rehabilitation; and improve self-efficacy. Among these, avoidance response was particularly prominent. This response was not only a psychological defence mechanism but also reflected patients’ profound recognition of life’s fragility. It manifested their existential anxiety about life’s finitude and their attempts to rebuild meaning of life by focusing on the future. Meanwhile, some patients experienced philosophical life changes such as re-prioritizing life matters and adjusting their views on life and death. The enhancement of empathy enabled patients to put themselves in others’ shoes and actively help others through self-disclosure, thereby reconstructing their existential value, reflecting the existential transformation in PTG. However, from the time the patient was transferred to the ICU for treatment until 6th month after transfer out of ICU, avoidance reactions continued to exist. This may be because patients wished to avoid re-experiencing the pain or improve their hope for the future, and hence continued displaying avoidance. Consequently, the need for information has always existed since the patient was transferred to the ICU. Moreover, the effort to obtain this information has been phased into “early treatment situation, and later rehabilitation guidance.”

### Growth transition period

This refers to the period when patients, after ICU treatment, guide themselves out of the haze by actively accepting reality, interpreting the ICU experience, developing interests, replanning life, and other behaviours to achieve individual growth and produce new needs for self-repair. Among these findings, manifestations such as the reconstruction of life philosophy reflected individuals’ transition from fear of death to cherishing present life, demonstrating how patients could construct new meaning of life through re-examining life and death. It manifested in all patients with Trajectory 1, with a high degree of flexibility. However, in Trajectory 2, it only appeared in patients with metamorphosis who were relatively well prepared physically and mentally.

### Trajectory 2: compound dual-path PTG trajectory

In this study, the “collapse period—transitional recovery period” emerged as a necessary stage in the post-traumatic growth trajectories in the vast majority of patients. Subsequently, the dual pathway appeared, A portion of patients progressed into the Growth Transformation Phase, whereas the patients who showed the common characteristics of low cultural level, poor career stability, and a long disease course were stuck in the transitional recovery period, thereby forming two types of transformation and delayed-recovery type. Simultaneously, various psychological characteristics in the trauma response period were interspersed in the dual pathway, forming a compound dual-pathway PTG trajectory.

### Collapse period

This stage refers to the period when patients experienced drastic physical and mental breakdown in the face of a sudden ICU treatment event. None of the patients in this study had a history of ICU treatment. The sudden changes and their severity were far beyond the patients’ cognitive tolerance. Inherent cognition caused the patients to respond to the ICU with high, relatively subjective treatment expectations. Therefore, the huge reality gap induced original cognition fragmentation. The special treatment environment, self-perception of disease, and heavy economic burden not only trapped patients in negative emotions, such as fear and anxiety but also significantly increased their demand for social support and humanistic care from relatives and medical staff. Simultaneously, when patients were poorly adapted to the environment, misunderstood the treatment operation, or could not understand humanistic care from medical details, negative stress reactions such as rejection and resistance were triggered.

### Transition recovery period

After the collapse period, although the physical and mental symptoms in patients have been relieved to a certain extent, some patients began to have optimistic expectations for recovery and actively attempted transition to a normal life. However, overall health problems such as physical decline, fear, anxiety, and nightmares remain. In addition, the uncertainty of the disease and the excessive worry of family members could hinder the patients’ reintegration into society to varying degrees, and patients are faced with new challenges such as the loss of their sense of identity due to continuous economic pressure, decreased self-protection, and adjustment of family roles.

## Discussion

This study, grounded in Interpretative Phenomenological Analysis (IPA), explores how ICU patients reconstruct their lifeworld and reframe the meaning of existence during the course of critical illness treatment.As a phenomenological approach with a psychological orientation, IPA facilitates an idiographic and in-depth exploration of individuals’ lived experiences and contextual meaning-making processes. The findings indicate that post-traumatic growth is reflected not only in behavioural and cognitive shifts, but more profoundly in patients’ deep reconstruction of life’s value and existential meaning. Through a double-hermeneutic lens, this study delves into how patients, when confronted with the threat of death, come to rethink the meaning of life and internalize this existential insight as a profound driving force behind post-traumatic growth.

Trajectory presents “two points” in common, and nurses should grasp the key time points and guide patients to accept “constant points.”

This study showed that the PTG trajectory of patients in ICU still retained “two-point” commonalities, namely “time point” and “constant point,” while fully displaying its heterogeneity. During the period of collapse, the patients experienced a moment of cognitive breakdown; their negative emotions and reactions were particularly prominent, and, combined with longitudinal observations, a significant continuation trend was observed. Saldana scholars pointed out that invariance is also an important change (Saldana, 2003) in longitudinal studies. Avoidance response, a common manifestation in patients, intersects with the two trajectories and becomes the “invariant point” of this study.

According to the broken world hypothesis (Janoff-Bulman, 1989), after experiencing a traumatic event, the original core belief system is affected, and an individual may develop a negative world hypothesis (Wagner et al., 2009). Based on this, PTG cognitive theory proposes that after the original world is broken, individuals rebuild their positive cognition of the world through cognitive (Shung-King et al., 2018) activities. Therefore, when patients enter a period of collapse and exhibit cognitive fragmentation and fear-avoidance reactions, core belief fragmentation and reconstruction are key points. Medical staff can guide patients to rationally analyse the causes of the disease, explain the disease and prognosis-related knowledge to help patients accept the reality and alleviate the cognitive impact, and guide patients to become familiar with treatment measures, enhancing their sense of security through skilled professional operations. Comforting and encouraging patients to express their emotions through writing, storytelling, and other means can reduce fear reactions. Maintaining patients’ dignity and arranging family visits enhance social support and other ways to promote patients’ active rumination and guide them to rebuild positive world cognition.

The appearance of PTSD-related symptoms, such as avoidance and intrusive thoughts, indicates that individuals try to cope with trauma through cognitive processing of traumatic information, and the generation of PTG will inevitably be accompanied by some degree of pain (Tedeschi & Calhoun, 1995). The PTG philosophy also emphasizes that growth is not the same as reducing or eliminating pain and that understanding the fullness of life and gains after trauma can truly transcend the pre-trauma state. This suggests that medical staff should guide patients to correctly define, objectively accept, and deeply understand the value of avoidance reactions. In the early stages, indirect psychological guidance, rather than direct and complete intervention, can be used to alleviate patients’ negative stress responses and negative emotions with the help of cognitive reconstruction and indirectly soothe patients’ avoidance responses. In the later stages, patients can be guided to actively explore the post-traumatic harvest, establish positive health beliefs, encourage participation in social life within their ability, and affirm the existing rehabilitation effects to protect their avoidance psychology and promote PTG.

Trajectory highlights personality differences, nursing staff should have insight into patients’ period characteristics and care for individual self-healing ability.

Through longitudinal exploration, this study draws the journey track in patients with PTG in ICU and clarifies the timing for clinically personalized interventions and the characteristics of ICU patients’ phased needs. The results showed that most patients were sensitive and vulnerable during the collapse period and had a significant need for information and humanistic care. Medical staff needed to intervene and guide the entire process. Nursing staff should pay attention to the protection of patients’ privacy details, inform them of the significance of treatment operation in a timely manner to help patients better adapt to the environment, and understand the treatment, which should be used in the operation to soothe their sensitive emotions and narrow the distance between nurses and patients. The inability to understand one’s own treatment and prognosis is an important reason for fear and anxiety. Medical staff may conduct a comprehensive assessment of the patient’s condition and, in consultation with family members, appropriately address and respond to the patient’s information needs. After patients enter the transitional recovery period, the frequency and intensity of intervention and guidance from medical staff should depend on the recovery of the patients. However, the overall trend is decreasing. Since the information needs of patients mainly involve rehabilitation guidance, medical staff can provide rehabilitation information to patients in the form of WeChat tweets, popular science videos, and the establishment of rehabilitation discussion groups.

Bonanno’s potential trauma longitudinal trajectory theory (Bonanno, 2004) proposes that individuals with mental resilience and self-healing abilities should be carefully intervened if they show good self-resilience. When patients smoothly enter the growth and transformation period and have self-healing needs, medical staff should gradually withdraw from interventional guidance, change from “facilitator” to “assistant,” actively provide post-hospital guidance for patients, and encourage them to take the initiative to carry out rehabilitation counselling based on their conditions. Patients experiencing delayed-recovery type who are stuck in the transitional recovery period should receive diversified rehabilitation guidance. Furthermore, nursing time should be appropriately extended to help patients cope scientifically with various problems.

Actively develop after-hospital ICU care based on MDT diagnosis and treatment mode and online and in-person linkage.

A multidisciplinary team (MDT) (Øvretveit, 1996) diagnosis and treatment model involves a team composed of two or more related disciplines that provide beneficial diagnosis and treatment suggestions for managing patients. Post-intensive care in the ICU has been reported not only to reduce the risk of PICS but also improve the corresponding symptoms and reduce mortality and medical needs (Friedman et al., 2022). However, this study found that relying solely on the telephone follow-up of nurses cannot meet the diversified and personalized needs of patients. Therefore, the continuation of care based on MDT diagnosis and treatment models is necessary. Post-hospital ICU diagnosis (Chen et al., 2022; Wan et al., 2021; Yang & Qiu, 2018) and treatment reported in practice have achieved good results but are still in the initial stage of development owing to time and space constraints. To overcome the limitations of traditional post-ICU continuous care in terms of time and space, establishing a hybrid “in-person” post-ICU continuity of care system is recommended. The online component involves the use of digital tools such as teleconsultations, health monitoring applications, and video follow-ups to provide patients with efficient and convenient rehabilitation support. The in-person component includes regular outpatient follow-up visits, rehabilitation support group activities, and community health services, with an emphasis on face-to-face communication and on-site interventions. Online services enhance the accessibility and responsiveness of care delivery, while in-person services strengthen interpersonal interaction and improve the accuracy of in-person assessments. By complementing each other, the integration of online and in-person services promotes the integrity and continuity of post-ICU nursing care. Medical staff should encourage family members to participate in extended after-hospital care, provide scientific and social support to patients, create a good rehabilitation environment, and help patients return to society.

This study has some limitations that should be acknowledged. The sample in this study was obtained from only one medical centre, and the time was limited. In the future, a long-term, multicenter, combined mixed research method can be used to draw the portrait of the intervention population and construct personalized intervention programs to improve the quality of life in patients in ICU after hospitalization.

## Conclusion

This study conducted four rounds of in-depth longitudinal interviews with 21 ICU patients over six months, exploring two distinct trajectories of post-traumatic growth (PTG). These trajectories were systematically examined across four phases and fifteen themes. The findings highlight key intervention points and phase-specific psychological characteristics in the PTG process among ICU patients, offering empirical support for the development of individualized, time-structured clinical interventions. The PTG trajectories exhibited both common features and individual differences: commonalities included key moments in trauma processing and the ongoing influence of avoidance mechanisms, while individual differences were reflected in the differentiation of growth paths, potentially shaped by factors such as educational background and levels of social support. Methodologically, this study combines a positive psychology perspective with longitudinal qualitative methods, thereby extending the application of PTG theory to the psychological well-being of ICU patients and highlighting the opportunities for psychological growth that emerge from the ICU experience. The findings suggest that healthcare professionals should identify key intervention points and guide patients in acknowledging and accepting avoidance responses. Additionally, care should be adapted according to patients’ temporal psychological characteristics to support their self-healing abilities. Furthermore, the establishment of an MDT-based, integrated online and in-person ICU aftercare model is proposed as a long-term development goal. Given the limited sample size and relatively short follow-up period, future research should adopt multi-centre, long-term designs to further validate these findings and strengthen the theoretical and practical foundations for post-discharge psychological interventions for ICU survivors.

## Data Availability

The participants of this study did not give written consent for their data to be shared publicly, so due to the sensitive nature of the research supporting data is not available.
